# *Podravka* and *Slavonka* Varieties of Pepper Seeds (*Capsicum annuum* L.) as a New Source of Highly Nutritional Edible Oil

**DOI:** 10.3390/foods9091262

**Published:** 2020-09-09

**Authors:** Tanja Cvetković, Jasmina Ranilović, Davorka Gajari, Helena Tomić-Obrdalj, Drago Šubarić, Tihomir Moslavac, Ana-Marija Cikoš, Stela Jokić

**Affiliations:** 1 Research and Development, Podravka Ltd., Ante Starčevića 32, 48000 Koprivnica, Croatia; jasmina.ranilovic@podravka.hr (J.R.); davorka.gajari@podravka.hr (D.G.); helena.tomic-obrdalj@podravka.hr (H.T.-O.); 2 Faculty of Food Technology Osijek, Josip Juraj Strossmayer University of Osijek, Franje Kuhača 20, 31000 Osijek, Croatia; dsubaric@ptfos.hr (D.Š.); tihomir.moslavac@ptfos.hr (T.M.); acikos@ptfos.hr (A.-M.C.)

**Keywords:** pepper seed, *Capsicum annuum*, oil, quality, bioactive compounds, sensory analysis

## Abstract

The aim of this study was to evaluate Croatian pepper seed varieties (*Podravka* and *Slavonka*) as a new source of added value ingredients. Pepper seed is mostly considered a by-product. For the first time, the pepper (*Capsicum annuum* L.) seeds of the *Slavonka* and *Podravka* varieties were examined as the source material for oil production by the two methods of extraction: cold pressing (CP) and supercritical CO_2_ extraction (SC-CO_2_). Further, fatty acid profile, tocopherols, and sensory analysis of the oils were examined, as well as the chemical characteristics and antioxidant potential of seed flour. The antioxidant potential of pepper seed flour was different between varieties (*Podravka* 107 antioxidant unit (AU); *Slavonka* 70 antioxidant unit (AU)). The *Podravka* variety pepper seed oil has shown higher **γ**-tocopherol content (CP 80.1 mg/100 g; SC-CO_2_ extraction 65.3 mg/100 g) than the *Slavonka* variety (CP 65.3 mg/100 g; SC-CO_2_ extraction 16.0 mg/100 g). According to the obtained results, cold pressing (CP) would be a more favourable method for pepper seed oil extraction, taking into account sensory evaluation and nutritional quality. The pepper seed oil has potential for culinary application with a nutritional quality comparable to vegetable oils of a higher price class.

## 1. Introduction

A fast-growing world population, deficiencies in feed supply, and global bioenergy demands are the main reasons for the increasing amount of research into by-products and waste from agro-food processing. It has been found that up to 39% of food waste is generated during manufacturing, and 5% is generated within food distribution systems. So, the utilization of food processing waste is very important for a sustainable agricultural economy [1,2]. According to botanical classes, peppers as plant vegetables are within the *Solanaceae* family and the *Capsicum* genus. Within the genus, the *Capsicum annuum* L. species is the most cultivated one and there are a lot of varieties of *Capsicum annuum* L. [3]. Annual global production reached approximately 3.9 million tons (dried chillies and peppers) in 2016 [4]. Asia is the largest pepper producer, accounting for 70% of global production [5]. The pepper fruits (*Capsicum annuum* L.) in different forms, colour, taste, shape, and pungency, and be used fresh, for cooking, or processed into sauces, condiments, spreads, powders, and other products [4].

Available published research has shown that pepper seeds are a great source of high quality oil, which can be used in nutrition, as well in the pharmaceutical, chemical, and cosmetic industries, due to their capability of absorbing ultraviolet (UV) radiation [6]. The mentioned research has shown that pepper seeds (*Capsicum annuum*) could be considered a sustainable source of dietary fibres, which are present at a high percentage (60.96% of total dietary fibres).

El-Adaway and Taha [7] were the first to evaluate the chemical composition of oil and proteins from pepper seeds. They reported that pepper seeds (*Capsicum annuum*) are a good source of protein (24%) and oil (26%). Subsequently, da Silva et al. [8] confirmed that seeds have high antioxidant potential due to their content of polyphenols, carotenoids, and terpenes.

The oil obtained from the pepper seeds has a pleasant taste which can be compared to other edible oils such as peanut oil [9] and sunflower oil [10]. The oil colour is reddish yellow, indicating the presence of carotenoids. In terms of physicochemical characteristics, pepper seed oil is in the range of other edible oils [11].

Reports in the literature have shown that the oil content of pepper seeds in the *Capsicum* genus varied from 14.6% to 35.9% among cultivated species, as well as that seed oil content varied between seven *Capsicum annuum* varieties from 16.4% to 35.9% [3] and from 8.5% to 32.6% among varieties from different locations in Italy and Turkey [12].

Regarding fatty acid composition, the variation between the species and varieties was small. The predominant fatty acid in pepper seed oils of *Capsicum annuum* was linoleic acid, which accounted for between 69.5% and 74.7% in different varieties [12] and from 70.9 to 74.3% according to Koncsek [13].

Further, according to Chouaibi [14], the total phenolic compounds and γ-tocopherol contents of pepper seed oils (*Capsicum annuum*) are influenced by the extraction techniques, as well as their antioxidant activities.

*Podravka* and *Slavonka* are among the first Croatian varieties of peppers (*Capsicum annuum* L.) with the UPOV (International Union for the Protection of New Varieties of Plants) certificate, and they are also added to the List of Varieties of the Republic of Croatia [15].

The names of the varieties are associated with two regions in the north of Croatia suitable for growing peppers. *Podravka* and *Slavonka* are medium early varieties of peppers, suitable for processing in the industry, so they are mostly used in the production of chutney and other condiments.

The aim of this study was to examine the nutritional, chemical, and sensory quality of oil obtained from the *Podravka* and *Slavonka* varieties of pepper seeds (*Capsicum annuum* L.) by the two green extraction methods (CP—cold pressing; SC-CO_2_—supercritical CO_2_ extraction) at the laboratory level.

## 2. Materials and Methods

### 2.1. Materials

Dried pepper seeds (*Capsicum annuum* L.) of the *Podravka* and *Slavonka* varieties were obtained in September of 2018 from the plant Črešnjevica in the Podravina region (north-western part of Croatia).

**Figure 1:**
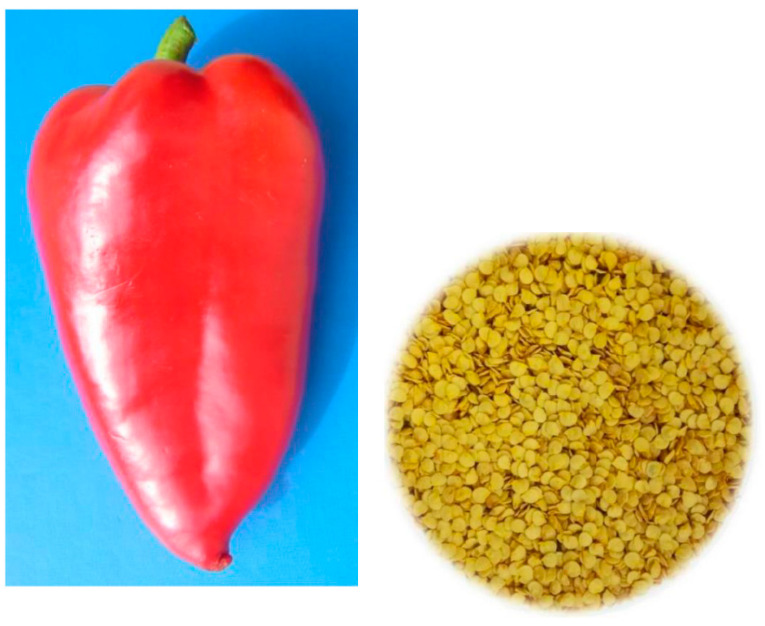
Fruit and seeds of the pepper (*Capsicum annuum* L.) variety *Podravka.*

**Figure 2:**
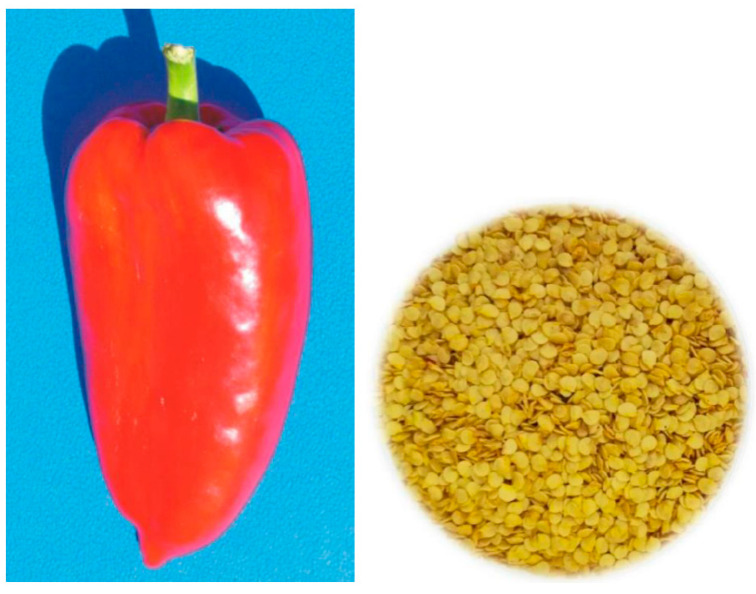
Fruit and seeds of the pepper (*Capsicum annuum* L.) variety *Slavonka.*

The pepper fruits (*Capsicum annuum* L.) of the *Podravka* and *Slavonka* varieties are conical oblong red-coloured fruits, with the average weight of 165 g (*Podravka*) and 150 g (*Slavonka*). There are no big differences in botanical performances between the two varieties of fruit [16]. The pepper fruits and seeds of the *Podravka* and *Slavonka* varieties used in this study are shown in Figure 1 and Figure 2.

Prior to analysis, the dried pepper seeds were ground into powder in an IKA A11 Basic laboratory mill. The purity of CO_2_ used for extraction was 99.97% (*w*/*w*) (Messer, Osijek, Croatia). Industry FAME mix 37 standard for fatty acid analysis was purchased from Restek (Bellefonte, PA, USA).

The oil obtained from both varieties and both extraction methods was packed into dark glasses and kept in a dry place at room temperature (20 °C) prior to the analysis.

All the chemicals and standards were purchased from Supelco (Bellefonte, PA, USA), Sigma (MO-St.Louis; PA-Allentown, and WY-Laramie, USA), Sigma-Aldrich (St. Louis, MO, USA; Germany; Oakville, ON, Canada), Honeywell (Charlotte, NC, USA), Nu-chek-prep (Elysian, MN, USA), Merck (St. Louis, MO, USA; Darmstadt, Germany), Acros Organics (Morris Plains, NJ, USA; Hangzhou, China; Geel, Belgium), Carlo Erba (Milano, Italy), Ultra Scientific (Bologna, Italy), HPC (Cunnersdorf, Germany), CHEM-LAB (Zedelgem, Belgium), VWR International (Radnor, PA, USA) and the reference material for determination of dietary fiber (CRM T2486QC, matrix is wheat flour) was purchased from FAPAS (York, UK).

### 2.2. Methods

#### 2.2.1. Supercritical CO_2_ (SC-CO_2_) Extraction of the Podravka and Slavonka Pepper Seeds

The experiment was performed in the SC-CO_2_ system explained in detail by Jokić et al. [17]. The extraction vessel is made from a stainless steel bar (AISI 304) O.D. 100 mm and height 500 mm. A stainless steel rod is drilled (center hole) with a Ø 40 mm bore for 400 mm, so the volume of the extractor is 500 mL. The upper inside part of the extraction cell is polished to plug well gaskets. The plug has a built-in filter element that should prevent withdrawal of material. The filter element has the ability to filter particles of 2 microns nominal and 10 microns absolute (Norman Ultraporous 4202T-6T-2M). High-pressure seamless tubes of dimensions 10 × 2 mm are connected to each other by Ermeto couplings (flat, knees, tees). The pressure extraction cell is controlled by two WIKA manometers (model 212.20) 60 MPa and one WIKA manometer (model 212.20) 4 MPa for pressure in the separator. The extraction cell is heated with a glass fiber electric heater, controlled by a centralized system and Solid State Relays (SSR). Temperature is controlled by means of a PID regulator set to a maximum temperature of 80 °C with lag delay compensation, due to the large mass of the extraction cell. Temperature measurements and regulation of the extraction cell are performed using an integrated temperature sensor within the cell and an additional temperature sensor measuring output gas temperature. The input CO_2_ line towards the extraction cell is preheated using a heat exchanger powered by a water heating system. The temperature is regulated using a standard PID regulator, taking into account the differential temperatures of water lines and output gas line. Pressure in the separator is regulated by means of an electromechanical solution for controlling the pressure valve, with a pressure sensor working as a feedback. The pump used to pressurize liquid CO_2_ is a Haskel^®^ MS-71. Liquid CO_2_ is precooled through the SS coil at −18 °C, cooled by an ethylene glycol/ethanol cooling bath. A check valve is located after the pump to prevent eventual CO_2_ flow disorders. Prior to the input extraction vessel, CO_2_ is preheated through a stainless steel double coil at the temperature of extraction. After the extraction vessel, the high pressure is reduced by a high-pressure valve (B-HV) to the desirable pressure. Valves and tubing are heated to a temperature of 0 °C due to high-pressure drop. The flow of CO_2_ is controlled through a Matheson FM-1050 (E800) flow meter.

In this study, ground dried pepper seeds, of the *Podravka* and *Slavonka* varieties, in the amount of 100 g, were placed into the extractor vessel. The extracts of both seed varieties were collected in previously weighed glass tubes. The extraction process took 3 h, and all of the raw material was exhausted. The amount of extract obtained after the defined time was established by weight, using a balance with a precision of ± 0.0001 g. The separator conditions were 15 bar and 25 °C. The SC-CO_2_ was performed at the extraction pressure of 300 bar and the temperature of 40 °C, at a mass flow rate of 2 kg/h. The oil yield obtained from *Podravka* seeds was 26.2%, and from *Slavonka* seeds, 25.5%.

#### 2.2.2. Cold Pressing of the Podravka and Slavonka Pepper Seeds

Cold pressed oil from pepper seeds, of the *Podravka* and *Slavonka* varieties, was obtained on a laboratory screw press (KOMET, screw oil expeller Ca 59 G, Germany). The following parameters were used: head press temperature of 93 °C, frequency of 22 Hz, and nozzle interior diameter of 15 mm. The temperature of the obtained crude oil was 38° C, the oil was left for sedimentation and the clear oil was then separated from the precipitate. The oil yield obtained by cold pressing from *Podravka* seeds was 22.7%, and 20.21% from *Slavonka* seeds.

#### 2.2.3. Pepper Seed Flour—Nutritional and Chemical Analysis

##### Determination of Energy Value

The value obtained by calculation and a note on the contents of fats, proteins, carbohydrates, and dietary fibre (where present), were expressed in kcal or kJ per 100 g of food in accordance with current legislation [18] on the nutritional labelling of food products.

##### Determination of Protein Content

Protein content was determined by the Dumas method [19], where an aliquot of a sample was subjected to combustion with oxygen using an automatic instrument (FP-528, LECO Inc., St Joseph, MI, USA), which measures the amount of total nitrogen present in the sample. The combustion temperature was 950 °C, while the time of the analysis process was 210 s. The nitrogen value obtained was multiplied by the specific conversion factor 6.25 related to the analysed matrix [20].

##### Determination of Fat Content

Warm acid hydrolysis was performed with 1:4 HCl: H_2_O solution, where 4 g of the sample was used during 60 min from the time of boiling. Filtration and drying of the filter were done in an oven at 70 °C. Hence, hot extraction with petroleum ether lasted for 3 h using the Soxhlet apparatus. At the end of the extraction, the solvent was evaporated and the residual fat was determined by gravimetric analysis [21].

##### Determination of Saturated Fatty Acid Content

The methyl esters obtained by transmethylation of the fat substance were analysed on the gas chromatograph 7890A (Agilent Technologies, Lake Forest, CA, USA) with a capillary column CP-Sil 88 (length 100 mm; diameter 0.25 mm; phase thickness 0.2 μm) (Agilent Technologies, Lake Forest, CA, USA), splitting ratio of 10:1, and a flame-ionization detector (Agilent, Lake Forest, CA, USA) with a sample volume of 1 µL. The starting temperature was 60 °C with holding time of 5 min. The oven temperature was increased by a rate of 15 °C/min to 165 °C, held for 1 min, then increased according to the rate of 2 °C/min to 225 °C, and held for 19 min. The carrier gas was helium (99.9999%) at the constant flow rate of 0.5 mL/min. Hydrogen flow was 40 mL/min, air flow was 450 mL/min, and the makeup gas flow (nitrogen) was 24 mL/min. Composition was determined using the response factors obtained from the analysis of a reference mixture of methyl esters of known composition, under conditions identical to those used for the sample. Saturated fatty acids were expressed with respect to the fat content of the sample [22].

##### Determination of Carbohydrate Content

The value of the carbohydrate content was obtained by calculation, considering the known values of fats, proteins, dietary fibres, water content, and ash [23].

##### Determination of Sugar Content

The sample was extracted in hot water and stirred for 30 min. Aqueous extract, in which internal standard (inositol for monosaccharides; fructose, glucose and trehalose for disaccharides; saccharose, lactose, maltose), was appropriately added, and was analysed after derivatization on the gas chromatograph 6890N (G1530N) (Agilent Technologies, Lake Forest, CA, USA) with a capillary column in fused phase-linked silica DB1 type (length: 30 m, internal diameter: 0.25 mm, phase thickness: 0.25 μm), a split injector (split ratio 15:1; split flow 15 mL/min), and a flame-ionization detector (FID; Agilent Technologies, Lake Forest, CA, USA), with the sample injection volume of 1 µL. The starting temperature was 160 °C with holding time for 2 min. Oven temperature was increased according to the rate of 12 °C/min to 280 °C, held for 8 min, then increased according to the rate of 20 °C/min to 290 °C, and held for 10 min. The carrier gas was helium (99.9999%) at the constant flow rate of 1 mL/min. Hydrogen flow was 40 mL/min, air flow was 450 mL/min, and the makeup gas flow (nitrogen) was 44 mL/min. The composition was determined using the calibration curve for sugars with concentrations ranging from 25 to 1000 mg/L [24].

##### Determination of Dietary Fibre Content

Dietary fibre was determined gravimetrically, after enzymatic digestion with a-amylase (100 °C water bath, to provide gelatinisation, hydrolysis, and depolymerisation of starch), protease (water bath at 60 °C to solubilise and depolymerise proteins), and amyloglucosidase (water bath 60 °C to hydrolyse the starch fragment to glucose). Samples were run in duplicates and treated with ethyl alcohol to precipitate soluble fibre and remove depolymerised protein and glucose (from starch). The total residue was washed with ethyl alcohol and acetone, dried, and then weighed. Residual proteins and ashes were then determined on the obtained residue. Total fibre content was provided by the weight of the residue after subtraction of the ash and residual protein content, all brought back to 100 g of sample. The samples with fat quantity of >10% were subjected to a preliminary treatment with an ethanol–petroleum ether mixture. The analytical value does not include soluble fibres, which are non-precipitable by ethanol (e.g., inulin, resistant maltodextrins, etc.), and which were detected using specific methods [25].

##### Determination of Sodium Content

An aliquot of homogenized sample was mineralized with 70% HNO_3_ at 120 °C in a heating block for 90 min. The obtained solution was then quantitatively brought to volume, filtered in a test tube, and appropriately diluted. Instrumental determination was performed using inductively coupled plasma-optical emission spectroscopy (ICP-OES). The method is validated and accredited in compliance with the EN ISO/IEC 17025 standard [26]. Instrument calibration was performed using certified standards (sodium standard, ultra scientific analytical solutions 10,000 µg/mL in water and diluted nitric acid). Control tests were conducted through international Proficiency Tests.

##### Determination of Water Content and Dry Matter

The sample was dried in an oven for 6–8 h (minimum time) at 103 °C, directly in the weighing bottle, without the addition of sand. The water content was then determined by weight and by the calculation of the residue [27].

##### Determination of Ash Content

An aliquot of sample (about 2–3 g) was weighed in a porcelain or platinum calibrated container (capsule or crucible) and was then incinerated at 550 °C until the organic substance completely combusted and a constant mass was reached. Ash content was recovered by determining the weight of the obtained residue [28].

##### Determination of Vitamin C Content

The sample was extracted with water in the presence of DI-dithiothreitol (DTT) in an ultrasonic bath. After appropriate dilution and filtration, final HPLC determination was performed with a diode array detector (DAD) (Varian Prostar 330). The determination was carried out by a liquid chromatrograph (Varian 9012Q) with chromatographic column C18 (5 µm; 3.0 × 250 mm). The mobile phase for HPLC-DAD detection was ascorbic buffer at pH = 5 composed of 0.3% anhydrous sodium acetate + 0.3% of Tetrabutylammonium hydrogen sulfate + 0.07% of Potassium chloride in water by HPLC and brought to pH = 5 with glacial acetic acid. Isocratic elution with 100% mobile phase was performed for 10 min (flux 0.6 mL/min). Due to interferences, the final determination was carried out in (LC-MS/MS). The sample was extracted with a solution of DI-dithiothreitol (DTT) in water (20 mg/L), in an ultrasonic bath for 2 min. The obtained extract was filtered using HV-PVDF filters (internal diameter 0.45 µm) and diluted in the DTT solution, depending on the concentration of the sample in the final extract, which was within the concentration range of the calibration curve. The final determination was carried out by a liquid chromatograph (Waters Acquity UPLC, The Netherlands) and a mass spectrometer (Applied Biosystem API 4000) with a 1.8 µm column, 2.1 × 150 mm (Acquity UPLC HSS T3). The mobile phases (Table 1) that were used were A: 0.002 M ammonium formate buffer with 0.1% (*w*/*v*) formic acid in water and 1% acetonitrile; B: 0.025% formic acid (*w*/*v*) in acetonitrile. The determination was performed according to the method described by Novakova et al. [29].

##### Determination of Total Polyphenols

The method applied was based on the extraction of polyphenols with methanol in an acid environment and subsequent reaction with the Folin–Ciocalteu reagent in the presence of a sodium bicarbonate solution. The produced blue colour has maximum absorption at λ = 765 nm and is proportional to the content of phenolic compounds. Polyphenols are determined by spectrophotometric analysis considering an external curve prepared from gallic acid [30,31,32].

##### HPLC Determination of Capsaicins

Total capsaicins were determined according to ISO 7543-2 [33] for the spices as follows; the sample was extracted in Soxhlet for 8 h with tetrahydrofuran (THF). The obtained extract was transferred quantitatively and diluted in THF in a volumetric flask. An aliquot of the extracts was filtered in a vial with PTFE filters (0.45 µm pores like Millipore or equivalent).

The final determination was carried out in the liquid chromatograph 1200 (Agilent Technologies, Lake Forest, CA, USA) with the column Phenomenex Gemini 5 µm C18 110 (250 × 3.0 mm) and the diode array detector 1100 (DAD; Agilent Technologies, Lake Forest, CA, USA). The mobile phases (Table 2) were 1% of acetic acid in water + 5% acetonitrile for A and 100% methanol for B. The conditions during the analysis were in accordance with ISO 7543-2 [33].

##### Determination of Antioxidant Power

Antioxidant power (*AP*) was determined according to the method described by Jung et al. [34] based on antioxidant capacity and antioxidant activity. Antioxidant capacity and reactivity were measured using Electron Spin Resonance (ESR) spectroscopy. The measurements discussed in this article were performed with the X-band ESR spectrometer Miniscope MS 300 (Magnettech, Germany) and the following technical parameters: 60 G sweep width, 100 Gain, 1 G modulation amplitude, 7 mW attenuation, 3365 G central field, 0.14 sec time constant. Antioxidative power (*AP*) is a parameter able to quantify both the reaction capacity and velocity of antioxidants. The test radical *DPPH* (2,2-diphenyl-1picryl-hydrazyl) was used as a detector molecule. At least 3 concentrations of the test sample were prepared in ethanol (99%) and added to DPPH to obtain an initial radical concentration of 0.1 mM. The signal intensity decay of each concentration of the test samples was recorded at different time intervals during the reaction until saturation was reached and all antioxidant-active molecules had reacted with the test radical. A first order kinetic was obtained from these intensities for each concentration set. The kinetic parameters were used to calculate reaction time (*tr*) and the static parameters were used to calculate characteristic weight (*wc*). Both parameters were used to calculate the *AP* by means of the following equation (Equation (1)), where *N* is the quantity of reduced free radicals characterized by free electrons (spins) or the quantity of applied concentration c of *DPPH*-spins, and *RA* is the reduction amplitude:

(1) $$AP=\frac{RA\times N\;\left(DPPH\right)}{tr\times wc}$$

For a direct comparison of different antioxidants, the *AP* method was standardized to the activity of vitamin C (Ascorbic acid). The antioxidative activity of a solution of 1 ppm of vitamin C was defined as an antioxidative unit (AU).

#### 2.2.4. Pepper Seed Oil—Chemical and Sensory Evaluation

##### Determination of Fatty Acid Composition

Preparation of fatty acid (FA) methyl esters was carried out according to the HRN EN ISO 12966-2:2011 [35] standard. The prepared FA methyl esters were analysed by gas chromatography according to HRN EN ISO [36]. The gas chromatograph 7890B (Agilent Technologies, Lake Forest, CA, USA) with a HP88 capillary column, 100 m long with a diameter of 0.25 mm, and a 0.20 microns thickness of the stationary phase (Restek, Bellefonte, PA, USA), a split injector (temperature 250 °C), and a flame-ionization detector (temperature 250 °C) were used with a sample volume of 1 µL. The starting temperature was 120 °C with holding time of 1 min. The oven temperature was increased according to the rate of 10 °C/min to 175 °C/min, held for 10 min, then increased according to the rate of 5 °C/min to 210 °C and held for 5 min, and after that according to the rate of 5 °C/min to 230 °C with holding for 5 min. The carrier gas was helium (99.9999%) at the constant flow rate of 2 mL/min. The hydrogen flow was 40 mL/min, air flow was 450 mL/min, and the makeup gas flow (nitrogen) was 30 mL/min. FA methyl esters in samples were identified by comparison with retention times of 37 FA methyl ester standards at the same conditions. Prior to standard and sample analysis, certified reference material (CRM) was prepared and analysed at the same conditions. The results were expressed as the percentage (%) of individual fatty acids to total fatty acids.

##### HPLC Determination of Tocopherols

The sample, placed in a volumetric flask, was dissolved and extracted in isopropanol in an ultrasonic water bath for 30 min. An obtained aliquot of the extract was filtered in vials with PTFE filters (0.45 μm). The final determination was carried out in the liquid chromatograph 1290 infinity series (Agilent Technologies, Lake Forest, CA, USA) with the column Kinetex 1.7u PFP 100A (150 × 2.10 mm), with a fluorometric detector 1290 infinity series (Agilent Technologies, Lake Forest, CA, USA). The mobile phases (Table 3) were 0.04% phosphoric acid in water + 5% acetonitrile for A and 100% acetonitrile for B. The analysis was performed according to the conditions described in the Commission Directive 2000/45/EC [37].

##### Sensory Evaluation

Quantitative descriptive analysis^®^ (QDA^®^) was used for the purposes of the sensory profile of pepper seed oil. The descriptive panel consisted of 6 female and 2 male participants aged between 35 and 48. Before the analysis, the panel had training sessions for the period of two months, two hours per week. The panel developed a lexicon of 11 sensory attributes for red pepper seed oil samples. Attributes were based on previous research regarding descriptive sensory assessment of vegetable oils (olive, safflower seed, cottonseed etc.) [38,39,40,41]. QDA^®^ was carried out in two replicates by using a 15-cm scale anchored by none on the left, and strong on the right. Samples were served in transparent glass cups covered by an aluminium lid, coded by a 3-digit number. Participants got the amount of 15 mL served at room temperature (24 °C). A slice of apple and carbonated water was used for neutralization. Samples were presented monadically to judges in a random order. Acceptability testing was performed by using a hedonic scale from 9 (like extremely) to 1 (dislike extremely) on a group of 60 untrained consumers. Profiling analyses and consumer testing were performed in EyeQuestion Data Collection v. 4.11.40 (EyeQuestion Software - Logic8 B.V. - Nieuwe Aamsestraat, The Netherlands).

##### Statistical Analysis

All instrumental analyses on Podravka and Slavonka seed varieties were measured in triplicate. The results are presented as the mean (n = 3) ± standard error. Statistical analysis for significant difference between varieties (Table 4) and extraction methods (Table 5 and Table 6) were performed by Student T-test and Two-way ANOVA with repetition and Tukey’s Honestly Significant Difference (HSD) test for significance between varieties and extraction methods. Statistical significance for all tests was set at *p* < 0.05.

Statistical analysis for sensory profiling data included descriptive statistics, Two-way ANOVA and Tukey’s HSD test for significance between varieties and extraction methods and multifactorial Principal component analysis (PCA). Statistical analysis for consumer data included descriptive statistics, One-way ANOVA and Tukey’s HSD test. Level of significance was set at *p* < 0.05.

Statistical analyses were performed in MS Excel 2016 (Microsoft Corporation, 2020) and EyeQuestion Data Collection v. 4.11.40 (EyeQuestion Software - Logic8 B.V. - Nieuwe Aamsestraat, The Netherlands).

## 3. Results and Discussion

### 3.1. Pepper Seed Flour—Nutritional and Chemical Analysis

Standard parameters such as moisture, fat, protein, and fibre content were determined in the pepper seeds, *Podravka* and *Slavonka* varieties (Table 4). The results showed that there were no big differences in the contents between these two varieties. The *Podravka* variety had higher initial moisture (8.80 g/100 g) and oil content (27.20 g/100 g) compared to the *Slavonka* variety, with lower initial moisture (8.50 g/100 g) and oil content (26.70 g/100 g). Consequently, dry matter of *Podravka* flour was 91.20 g/100 g, while for *Slavonka* flour it was 91.50 g/100 g. The protein content was determined by the Dumas method. The results showed slight differences in protein content among the pepper seeds of both varieties. The protein content was lower (16.50 g/100 g) in the *Slavonka* variety compared to the content found in the *Podravka* variety (16.70 g/100 g). Despite the differences, seeds of both varieties can be considered as a good source of protein. El-Adaway and Taha [7] found that *Capsicum annuum* seeds were a good source of protein (24.43%) and oil (25.91%), while Azabou et al. [6] found that *Capsicum annuum* seeds had lower values of protein (18.30%) and oil (11.04%). Hot pepper seeds were also shown to be a good source of protein due to the content of 21.29 ± 0.28 g/100 g found by Zou et al. [42]. These variations between protein and oil content occurred due to the differences in plant variety, climate, harvesting time, and ripening stage [43]. Some reports have shown variations in the oil content of pepper seeds in the *Capsicum* genus, as well as seed oil content between *Capsicum annuum* varieties. According to Jarret et al. [3], the means of the seed oil content among five cultivated species of *Capsicum* genus varied from 18.26% to 28.08% and between *Capsicum annuum* varieties from 21.06% to 28.08%. Matthaus et al. [12] investigated 10 samples of *Capsicum annuum* from different locations (Turkey, Italy) and found that the oil content varied in a wide range from 8.5 g/100 g to 32.6 g/100 g. Seed oil made of samples from Italy showed a higher oil content than those from Turkey. There were big differences in seed oil content between different varieties in *Capsicum annuum* species, which is not in accordance with the findings in this study. A previous study by Jarret et al. [3] showed that environment does not have a significant influence on seed oil content obtained from the same pepper variety of *Capsicum annuum*. They analysed the seed oil content of seven accessions from different growing seasons and locations, and found the average difference between samples of the same pepper variety to be only 2.4%.

Regarding the results of the nutritional potential of the pepper seed flour, it was shown that they possess a high energy value of 407 kcal/100 g for the *Podravka* variety and 404 kcal/100 g for the *Slavonka* variety (Table 4). The fat content of the *Podravka* seed flour was higher (27.20 g/100 g) with lower values of saturated fatty acids (3.18 g/100 g) than the fats present in the flour of *Slavonka* seeds (26.70 g/100 g), but with higher values of saturated fatty acids (3.89 g/100 g). Paired T-test showed significant difference in saturated fatty acid content between varieties (*p* ˂ 0.05). There are no significant differences in seed oil content between the *Podravka* and *Slavonka* varieties, which is not in agreement with the previous study [3,12].

Regarding carbohydrate content, the flour of *Slavonka* seeds contained 3.40 g/100 g with 3.02 g/100 g of sugars, while the flour of *Podravka* seeds had lower carbohydrate content 3.20 g/100 g with 3.16 g/100 g of sugars. Embaby and Mokhtar [10] found that the carbohydrate content in sweet pepper seeds is significantly higher than that observed in this study, with the amount of 56.28 ± 0.49% of dry weight. It can be explained by the difference in calculation due to the dietary fibre value included in the carbohydrate content.

The high amounts of fibres in the amount of 41.20 g/100 g for the flour of *Podravka* seeds and 42.10 g/100 g for the flour of *Slavonka* seeds indicate that the pepper seeds could be beneficial for human health due to the ability of fibres to prevent colon cancer, obesity, cardiovascular problems, and diabetes [44]. Compared with the amount of dietary fibres found in hot pepper seeds (38.76 g/100 g) by Zou et al. [42], seeds used in this study showed higher amounts of dietary fibres and they were also the main components found in *Podravka* and *Slavonka* seeds. The daily requirement of dietary fibre is 21–25 g for women and 30–38 g for men, depending on their age [45]. An investigation by Anderson et al., 2020 [46] showed that the unweighted mean intake values for total dietary fibre were 16.7 g/day for men and 15.6 g/day for women and concluded that normal fibre intake is below the recommended levels. Therefore, one of the main strategies identified by the World Health Organization is food fortification and this is an opportunity for the food industry to apply fibre in products and develop new fibre-rich products [47]. Dordevic et al. 2020 [48], stated that the addition of more than 1% of bamboo fibre in fruit jams significantly affected sensory properties and caused the product to not be accepted by consumers. This value was not enough to satisfy the requirement to be labelled as the source of fibre (at least 3%), according to the [49]. Due to the high value of dietary fibre, pepper seed flour could be an ingredient for the fortification of different products, like jams, sauces and soups, but further evaluation of their nutritional, textural, and sensory characteristics, influenced by the fortification, is needed.

The results for salt showed that the flour of *Podravka* seeds contained 5.05 g/100 g of salt, while the flour of *Slavonka* seeds had a higher value of 5.18 g/100 g. Ash content, when compared to the red pepper researched by Azabou et al. [6] was lower for both of the flours; *Podravka* flour contained 2.91 g/100 g, while *Slavonka* flour had 2.77 g/100 g. Hot pepper seeds also showed higher ash content (4.94 ± 0.14 g/100 g) [42], as well as sweet pepper seeds (4.88 ± 0.15 g/100 g) [10] compared to the seeds used in this study.

In this study, vitamin C was not detected in *Podravka* seed flour or in *Slavonka* seed flour. However, studies have shown that ascorbic acid is present in Jalapeno peppers and its by-products, including the seeds and placenta. Sandoval-Castro et al. [50] revealed the content of ascorbic acid ranging from 0.0394 ± 0.014 g/kg to 0.0496 ± 0.0008 g/kg. Silva et al. [51] investigated two different species of pepper seeds, Reus long pairal and sweet Italian, and they observed that the sweet Italian pepper seeds contained a higher amount of ascorbic acid (0.2304 ± 0.0011 g/kg). Total capsaicins were not detected, which indicates that the used pepper seeds were sweet, while Jalapeno peppers and their by-products were spicy, which has been proven in the study by Sandoval-Castro et al. [50], where the amount of total capsaicins ranged from 0.142 ± 0.123 g/kg to 0.292 ± 0.304 g/kg. The capsaicionid content can differ between the cultivars, but the maturity stage plays an important role as well. Capsaicins are developed during maturation but also can be lost due to peroxidase activity [52].

It is well known that polyphenols contribute to the quality and the nutritional value of food, so their content, as well as their antioxidant power, were determined in this study. Polyphenols have the ability to reduce free radicals and prevent the damage they cause by providing hydrogen, quenching singlet oxygen and acting as metal chelators due to the hydroxyl groups. This study provides the confirmation of health benefits of pepper seeds due to the presence of phenolic compounds. The literature covers research on different species of pepper seeds, such as red pepper seeds, where the polyphenol content was 21.50 mg GAE g^−1^ of seed extract according to Azabou et al. [6], while Sim and Sil [53] recorded that the polyphenol content was 29.10 ± 0.18 mg GAE g^−1^ of pepper seeds. Hot peppers showed different polyphenol content when compared to red peppers. According to Gurnani et al. [54], red chilli pepper seeds contained from 7.95 mg GAE g^−1^ to 26.15 mg GAE g^−1^ of phenolic compounds, while Jalapeno peppers had slightly lower values of polyphenols (10.01 ± 0.61 mg GAE g^−1^ to 13.09 ± 0.98 mg GAE g^−1^) [50]. In this study, *Podravka* pepper seeds contained 158.20 mg/100 g of polyphenols, while *Slavonka* pepper seeds contained lower quantities (149.90 mg/100 g) (Table 4). This can be explained by the fact that polyphenol content depends on the pepper cultivar [55]. The results obtained by Marin et al. [56] showed that sweet peppers had a very rich profile of polyphenols and they have observed that the maturity stage from green to red decreased the total phenolic content in peppers.

The determination of antioxidant power (AP) is performed by ESR spectroscopy and it is based on the 1,1-diphenyl-2-picryl-hydrazil (DPPH) method, with the major difference that both the antioxidant capacity and the antioxidant activity are used to qualify an antioxidant [34].

The reaction time *t_r_* is a measure of the reactivity of the specific antioxidant and the characteristic weight *w_c_* reflects the capacity of the antioxidant. Both parameters are used to calculate AP [34]. The results for antioxidant power determined with the DPPH radical assay showed a correlation with the concentration of phenolic compounds. Apart from phenolic compounds, vitamin E and unsaturated fatty acids can also influence antioxidant capacity [14]. Antioxidant power for *Podravka* pepper seed flour was significantly higher (107 AU), compared to the *Slavonka* pepper seed flour (70 AU), which is in accordance with the polyphenol content found in these two flours. This is in correlation with lower reaction time (*t_r_*) of *Podravka* seed flour (1.01 min) compared to the *Slavonka* seed flour (1.10 min). The shorter the reaction time, the lower the oxidation state of polyphenols and the higher their antioxidative power.

A similar observation was presented in the study by Sim and Sil [53], where they have suggested that phenolic compounds were responsible for the antioxidant activities of red pepper pericarp and red pepper seeds. Silva et al. [51] observed that antioxidant activity was concentration-dependent for both of the analysed seeds, ‘‘sweet Italian’’ and ‘‘Reus long parial”. According to Sousa et al. [57], the antioxidant potential of pepper seeds is the consequence of the protection of lipids stored in seeds, as well as ensuring their viability in the presence of high oxygen concentrations during the germination process. Chouaibi et al. [14] revealed that the applied extraction method can have a significant influence on the oxidative stability of the oil. The oils produced by SC-CO_2_ extraction and cold pressing had better stability than the oil extracted by Soxhlet, considering the content of phenolic compounds. Statistical analysis of instrumental data obtained by the Paired T-test showed significant difference in saturated fatty acid content, sugars, and antioxidant power (*p* < 0.05). The Podravka variety (P1) has higher content of fatty acids, sugars, and higher antioxidant power than the Slavonka variety (P2). Differences in other measured parameters are not significant.

### 3.2. Pepper Seed Oil—Chemical Analysis

The determined fatty acid profile for the oils of *Podravka* and *Slavonka* seeds produced by SC-CO_2_ extraction, as well by cold pressing (CP), revealed the same trend among the detected fatty acids. Of the saturated fatty acids, palmitic and steric acid were determined, while oleic and linoleic acid were the main unsaturated fatty acids. Palmitic acid (C16:0) was dominant among the saturated fatty acids (SFA), with the highest amount present in the *Podravka* oil produced by SC-CO_2_ extraction (11.89%), followed by stearic acid (C18:0) (3.25%). It was found that linoleic acid (C18:2) was dominant in the oils produced from both seed varieties, using both of the methods. However, the cold pressed oil produced from *Slavonka* seeds had the highest amount of C18:2 (77.69%), while its content in the oil produced by SC-CO_2_ extraction was the lowest compared to the other obtained oils (74.01%) (Table 5). Significant amounts of oleic acid (C18:1) were found in the cold pressed oil of *Podravka* seeds (10.41%), while in other oils it was present in lower amounts; 8.35% for the oil of *Slavonka* seeds produced by SC-CO_2_ extraction, 8.43% for the cold pressed oil of *Slavonka* seeds, and 8.84% for the oil of *Podravka* seeds produced by SC-CO_2_ extraction. These findings were in accordance with the study of Azabou et al. [6], where they have found the dominance of C18:2 (70.93 ± 0.84) among the polyunsaturated fatty acids (PUFA); the dominant monounsaturated fatty acid (MUFA) was C18:1 (12.18 ± 0.22%), while C16:0 and C18:0 were the dominant SFAs (11.90 ± 0.33 and 3.54 ± 0.20). Konscek et al. [13] reported that variety and growing season have no influence on the fatty acid composition in spice pepper seed oil. The results showed small variations in the amount of determined fatty acid C18:2 (70.79–74.31%), C18:1 (7.9–9.11%), C18:0 (3.10–3.75%), and C16:0 (11.08–12.2%). A similar conclusion was found according to Matthaus et al. [12]; the variation in linoleic acid (C18:2) between varieties of *Capsicum annuum* was small (69.5 g/100g–74.7 g/100g), as well as for the other determined fatty acids (C16:0/10.7–14.2 g/100g, C18:0/2.5–4.1 g/100g and C18:1/8.9–12.5 g/100g). Reddy and Sarojini [9] found that chili (*Capsicum annuum*) seed oil contained 16.4.g/100 g of palmitic acid, 2.2 g/100 g of stearic acid, 10.9.g/100 g of oleic acid, and 70.6 g/100 g of linoleic acid.

Embaby and Mokhtar [10] suggested that the sweet pepper seed oil can be used as an edible cooking or salad oil due to the presence of high amounts of C18:2 (71.55%). In their study, they have obtained similar values of fatty acids to those determined in this study (C16:0/12.32%; C18:0/3.15%; C18:1/12.98%; C18:2/71.55%). These findings are in accordance with the fact that the fatty acid composition of the species within a genus is generally similar to Jarret et al. [3].

Linoleic acid is essential in the diet, as it is incorporated into cell membranes and it is involved in the synthesis of compounds that are responsible for regulating blood pressure, as well as for inflammatory response. In addition, PUFAs are considered beneficial for health due to their ability to reduce total cholesterol and body fat [6].

Konscek et al. [13] have showed that 10 g of pepper seed oil as salad oil can cover 70–74% of the suggested beneficial minimum daily intake and, according to the Commission (EU) No 1924/2006 [49] and 432/2012 [58], the following statements can be used for oils: “Linoleic acid (LA) contributes to the maintenance of normal blood cholesterol levels. The beneficial effect is obtained with a daily intake of 10 g of LA” and “Replacing saturated fats with unsaturated fats in the diet contributes to the maintenance of normal blood cholesterol levels.”

Li et al. [59] obtained red pepper seed oil by SC-CO_2_ extraction with and without ethanol as a co-solvent. The results showed that the oil obtained by SC-CO_2_ extraction without ethanol contained a lower amount of C18:2 (46.49%) compared to the one obtained with ethanol as a co-solvent (48.20%), but it was the dominant fatty acid in both of the oils. Chouaibi et al. [14] obtained similar results for C18:2 content to those in this study. The red pepper seed cold pressed oil had lower content of C18:2 (73.65%) compared to the *Podravka and Slavonka* variety seed oils used in this study (C18:2 75.37–77.69%), but the oil produced by SC-CO_2_ extraction had very similar content of C18:2 (76.26%) to the *Podravka* seed oil used in this study C18:2 (76.01%). Comparison with other oils such as soybean, sunflower, and peanut oils revealed that pepper seed oil had higher amounts of palmitic and linoleic acid [60,61,62] and it is classified among linoleic acid-rich edible oils.

Fatty acid compositions for both varieties and both methods are in accordance with previous results for *Capsicum annuum* L. species as well as linoleic acid (C18:2) content. The results showed that the variety of seeds or extraction methods had no effect on fatty acid content. Two-way ANOVA with repeated measurements indicated that there are significant differences in the content of oleic fatty acid between both varieties and extraction methods (*p* < 0.05). There was no significant influence of the variety or the extraction method on other measured fatty acids. The results showed that the variety of seeds, as well as the extraction methods, had no effect on the most dominant linoleic acid content (C18:2) of pepper seed oil.

Hence, among the analysed tocopherols, only γ-tocopherol was detected; α-tocopherol, α-tocopherol acetate, β-tocopherol, δ-tocopherol, and α-tocotrienol were not detected in this study (Table 6). The results showed that γ-tocopherol is the most dominant vitamin E-active compound in pepper seed oil. The highest amount of γ-tocopherol was found in the oil of *Podravka* pepper seeds produced by cold pressing (80.1 mg/100 g), followed by the amount in the oil of *Slavonka* pepper seeds also produced by cold pressing (65.3 mg/100g). Generally, the oil of *Podravka* pepper seeds showed a higher amount of γ-tocopherol compared to the oil of *Slavonka* pepper seeds. Matthaus and Őzcan [12] analysed 10 different varieties of pepper seed oil and showed that γ-tocopherol was also dominant in all of the samples, but other tocopherols were also detected (α-tocopherol, δ-tocopherol and β-tocotrienol). The amount varied from 306.6 mg/kg (Italy, red) to 602.6 mg/kg (Anamur table, bitter) which is a lower concentration compared to the cold pressed oils obtained in this study from both seed varieties. α-tocopherol, δ-tocopherol and β-tocotrienol were present in a much lower amount (49.8 ± 0.8 mg/kg, 2.8 ± 0.5 mg/kg and 6.1 ± 0.5 mg/kg), while these were not detected in the oils obtained in this study. Moreover, they noticed that the content of vitamin E-active compounds was higher in the pepper seed oil obtained from bitter pepper seeds, when compared to those with sweet taste. In addition, Koncsek et al. [13] did not detect β-tocopherol and δ-tocopherol in cold pressed spice pepper seed oil, while the amounts of α-tocopherol (13.51–16.41 mg/100 g) were significantly lower compared to the dominant γ-tocopherol (57.85–83.57 mg/100 g). They found a big variation in γ-tocopherol content of pepper seed oil among different varieties and harvest seasons and concluded that γ-tocopherol content in pepper seed oil was significantly affected by raw material variety and the growing season factors and their interactions. The results in this study match the previous results. There is significant variation in γ-tocopherol content between cold pressed pepper seed oil of *Podravka* and *Slavonka* varieties (80.1–65.3 mg/100g), as well as between *Podravka* and *Slavonka* pepper seed oil obtained by SC-CO_2_ (44.7–16.0 mg/kg). The obtained results for γ-tocopherol content showed variations between extraction methods and varieties too. γ-tocopherol content in *Podravka* and *Slavonka* seed oil obtained by cold pressing were higher than γ-tocopherol content obtained by SC-CO_2_ for both varieties. These values do not match those found by Chouaibi et al. [14] for γ-tocopherol content in pepper seed oil obtained by SC-CO_2_ extraction (130.56 mg/kg) and γ-tocopherol content of cold pressed pepper seed oil (113.24 mg/kg). There are significant variations in γ-tocopherol content between two extraction methods inside one variety. When it comes to vitamin E, there are significant differences and a noticeable influence of the variety and the extraction method on the content of vitamin E (tocoph. equi.).

According to the study performed by Gunstone [60], γ-tocopherol protected the cold pressed spice pepper seed oils from autooxidation and provided oxidative stability to the oils. This was confirmed in the study by Yang et al. [63], where it was shown that capsaicins and tocopherols protected pepper oil from thermal oxidation during frying.

This study showed significant differences in γ-tocopherol content between two varieties of pepper seed oil, as well as between two extraction methods.

### 3.3. Pepper Seed Oil—Sensory Evaluation

Sensory profile of the samples was described by the 11 attributes (Table 7) of taste, odour, and appearance. Sensory evaluation was performed on the pepper seed oil produced by the CP and SC-CO_2_ extraction methods. Overall, the results did not show significant differences between oil samples. Both obtained pepper seed oils had dark orange-red colour, pleasant odour, and the characteristic taste of pepper. When compared to the obtained oils of both the *Podravka (P1*) and *Slavonka (P2*) varieties, it was noticed that the pepper seed oil produced by cold pressing (CP) had more characteristic flavour than the one produced by SC-CO_2_ extraction. The sensory properties of the pepper seed oil confirmed that this oil could be applied in the food industry as salad oil or cooking oil for the preparation of spreads, soups, stews, and meat. Koncsek et al. [13] evaluated the technological and nutritional properties of the cold pressed spice pepper seed oil and concluded that it can be used in many food industries, as cooking and salad oil, due to the high content of phytonutrients.

The sensory profile of two different oil extraction techniques on two varieties of pepper seeds (Table 8) results in a slight difference in colour; the difference is significant between cold pressed oil samples (P1-CP) and supercritical CO_2_ extraction processed samples (P1-SC-CO_2_) without a big impact of variety (*p* < 0.01). Samples P1-CP and P2-CP have a slightly darker, more intense orange colour than P1-SC-CO_2_ and P2-SC-CO_2_. Turbidity shows a difference in variety; the P1 in both extraction methods is slightly clearer than the P2 samples, but not significantly.

**Figure 3:**
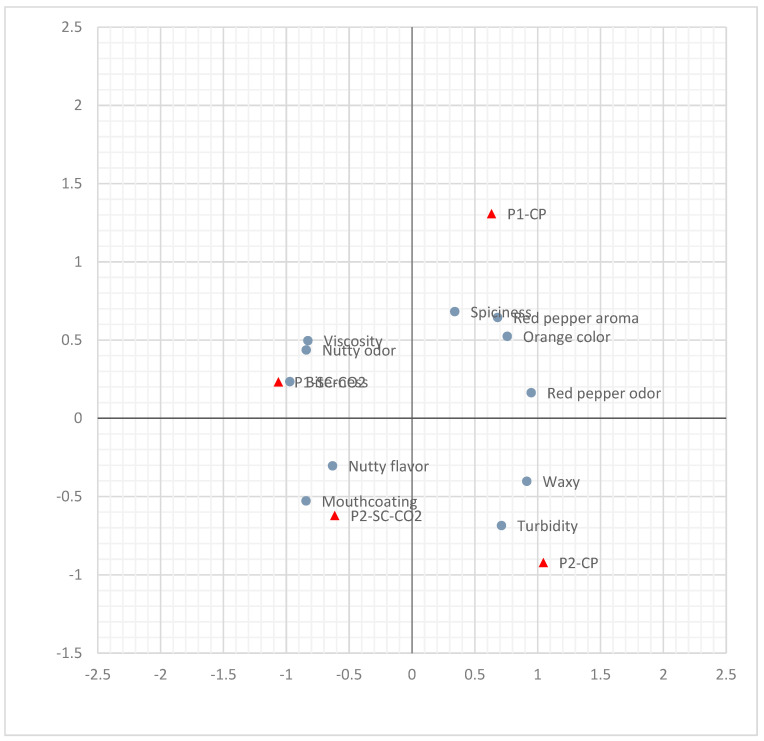
PCA analysis of four samples of pepper seed oil obtained by different extraction processes.

Odour is characteristic for red pepper, more intense in P1-CP and P2-CP than P1-SC-CO_2_ and P2-SC-CO_2_. Nutty odour is also mildly present; differences between varieties and extraction methods are not significant. Bitterness is very slightly present, without differences between varieties and extraction methods. Red pepper aroma is more expressed in the P1 variety, significantly between extraction methods for the P1 variety (*p* < 0.05). Nutty flavour is almost equally present in both varieties and extraction methods. Spiciness is more present in the P1-CP sample, but differences are not significant. There is only a slightly present waxy taste. Viscosity is typical of oils. Mouthcoating is also present to some extent, but mildly; however, there is a difference between extraction methods for the P1 variety (*p* < 0.05). A common characteristic of different seed oils is mild nutty flavour and spiciness, as shown in Aydeniz et al. [38]; in this study, those characteristics are more strongly expressed for CP oil samples. Despite the differences in the type of pepper seed oil samples, the negative attributes like bitterness of the SC-CO_2_ oil samples were similar in intensity and the waxy taste of all oil samples was less expressed, in comparison with Yılmaz et al. [64]. Multifactor PCA analysis (Figure 3) shows that PC1 participates with 62.23%, and PC2 24.35% in the variability of samples. The sample P1-CP is characterized by a red pepper odour and aroma, and intense colour. P2-CP is characterized by turbidity and waxiness. P1-SC-CO_2_ is characterized by a nutty odour, viscosity, and bitterness. P2-SC-CO_2_ is characterized by a nutty flavour and mouthcoating.

The obtained results from this study also show that oils produced by cold pressing have better acceptability (Table 9) than samples produced by SC-CO_2_. The variety of pepper seed oil Podravka (P1) has significantly better acceptability than the variety of pepper seed oil Slavonka P2 obtained by both techniques (*p* < 0.05). In comparison with Yılmaz et al. [64], hedonic test results of P1-CP showed high acceptance (8.1), which indicates a pepper oil with high potential for commercial use.

According to the sensory analysis, it can be concluded that the Podravka variety (P1) of pepper seed oil obtained by cold pressing has the most intense sensory profile, due to the darker orange colour, more intense red pepper odour and aroma, and pleasant spiciness, and it is the most acceptable according to the consumer acceptability test.

## 4. Conclusions

Pepper seed oil extraction by the cold pressing method showed to be a better technique for the extraction of natural antioxidants, which were present in the seeds; it was also better in the sensory analysis.

According to the results of the sensory evaluation and consumer testing, the pepper seed oil can be used as a valuable edible oil. Further investigations based on pepper seed oil culinary evaluations, as well as on added value product development, should be made in the future.

Due to the nutritional value of pepper seed flour, further investigations on product applications and development should be made as well. Pepper seed flour could be considered an added value ingredient for the development of new products.

We assume that the results from this study would contribute to the growing demand for recycling food by-products into new added-value products, and help food producers to achieve higher efficiency of production through the development of innovative and/or new ingredient(s), food product(s), and/or culinary applications.

## Figures and Tables

**Table 1:** Elution program for Vitamin C.

Time (min)	Mobile Phase A(%)	Mobile Phase B(%)	Flux (mL/min)
0	100	0	0.4
2.5	100	0	0.4
3.0	80	20	0.4
5.0	60	40	0.4
5.20	0	100	0.4
5.80	0	100	0.4
6.0	100	0	0.4
7.0	100	0	0.4

**Table 2:** Elution program for capsaicins.

Time (min)	Mobile Phase A(%)	Mobile Phase B(%)	Flux (mL/min)
0	40	60	0.650
17.50	30	70	0.650
18.00	0	100	0.650
Stop time:	22.50 min		
Post time:	4.00 min		

**Table 3:** Elution program for tocopherols.

Time (min)	Mobile Phase A(%)	Mobile Phase B(%)	Flux (mL/min)
0	50	50	0.6
5.0	35	65	0.6
15.0	25	75	0.6
15.1	0	100	0.6
16.0	0	100	1.0
Stop time:	19 min		
Post time:	3.00 min		

**Table 4:** The nutritional and chemical analysis of the *Podravka* and *Slavonka* pepper seed flour.

		*FLOUR*	
Parameters	Unit	*Podravka*	*Slavonka*
ENERGY	kcal/kJ/100 g	407/1674	404/1663
PROTEINS (Dumas method)	g/100 g	16.70 ± 0.37 a	16.50 ± 0.17 a
FATS	g/100 g	27.20 ± 0.54 a	26.70 ± 0.79 a
SATURATED FATTY ACIDS	g/100 g	3.18 ± 0.06 b	3.89 ± 0.08 a
CARBOHYDRATES	g/100 g	3.20 ± 0.008 a	3.40 ± 0.22 a
SUGARS	g/100 g	3.16 ± 0.05 a	3.02 ± 0.05 b
FIBRE	g/100 g	41.20 ± 0.88 a	42.10 ± 1.61 a
SODIUM	g/100 g	2.02 ± 0.01 a	2.07 ± 0.07 a
SALT (Nax2,5)	g/100 g	5.05 ± 0.03 a	5.18 ± 0.16 a
MOISTURE	g/100 g	8.80 ± 0.22 a	8.50 ± 0.17 a
ASH	g/100 g	2.91 ± 0.12 a	2.77 ± 0.09 a
VITAMIN C	mg/100 g	<LQ	<LQ
POLYPHENOLS	mg/100 g	158.20 ± 3.6 a	149.90 ± 2.69 a
TOTAL CAPSAICINS	g/100 g	<LQ	<LQ
DRY MATTER	g/100 g	91.20 ± 1.0 a	91.50 ± 1.04 a
ANTIOXIDANT POWER	AU	107.00 ± 4.55 a	70.00 ± 3.74 b
REACTION TIME	min	1.01 ± 0.06 a	1.10 ± 0.11 a
WEIGHT	mg	3.056 ± 0.09 a	4.271 ± 0.09 a

Values are mean ± SD of three replicates; different letters within the same row indicate significant differences (*p* < 0.05).

**Table 5:** The determination of fatty acid content in pepper seed oils, *Slavonka* and *Podravka* varieties, obtained by SC-CO_2_ extraction and cold pressing.

Fatty Acid (%)	SC-CO_2_ Extraction	Cold Pressing	SC-CO_2_ Extraction	Cold Pressing
	*Slavonka* Variety		*Podravka* Variety	
Palmitic acid (C16:0)	10.96 ± 0.45 a	10.89 ± 0.415 a	11.89 ± 0.205 a	10.84 ± 0.24 a
Stearic acid (C18:0)	3.01 ± 0.025 a	2.97 ± 0.13 a	3.25 ± 0.16 a	3.37 ± 0.095 a
Oleic acid (C18:1)	8.35 ± 0.095 d	8.43 ± 0.17 c	8.84 ± 0.18 b	10.41 ± 0.44 a
Linoleic acid (C18:2)	74.01 ± 0.475 a	77.69 ± 0.135 a	76.01 ± 0.21 a	75.37 ± 0.285 a

Values are mean ± SD of three replicates; different letters within the same row indicate significant differences (*p* < 0.05).

**Table 6:** Determination of tocopherols of the *Podravka* and *Slavonka* pepper seed oil obtained by SC-CO_2_ extraction and cold pressing.

		*OILS*			
Parameters	Unit	Cold Pressed Oil of Podravka Pepper Seeds	Cold Pressed Oil of Slavonka Pepper Seeds	SC-CO_2_ Oil of Podravka Pepper Seeds	SC-CO_2_ Oil of Slavonka Pepper Seeds
α-tocopherol	mg/100 g	<LQ^1^	<LQ	<LQ	<LQ
α-tocopherol acetate	mg/100 g	<LQ	<LQ	<LQ	<LQ
β-tocopherol	mg/100 g	<LQ	<LQ	<LQ	<LQ
δ-tocopherol	mg/100 g	<LQ	<LQ	<LQ	<LQ
γ-tocopherol	mg/100 g	80.1 ± 1.47 a	65.3 ± 2.06 b	44.7 ± 0.64 c	16.0 ± 0.78 d
α-tocotrienol	mg/100 g	<LQ	<LQ	<LQ	<LQ
Vitamin E (tocoph. Equi.)	mg/100 g	8.01 ± 0.15 a	6.53 ± 0.21 b	4.47 ± 0.06 c	1.60 ± 0.08 d
Vitamin E (UI^2^)	UI/100 g	11.90 ± 0.22 a	9.73 ± 0.31 b	6.66 ± 0.10 c	2.38 ± 0.12 d

Values are mean ± SD of three replicates; different letters within the same row indicate significant differences (*p* < 0.05). ^1^ limit of quantification (LQ) ^2^ international unit (UI).

**Table 7:** Description of attributes used in the sensory analysis of pepper seed oils by the trained panel.

Attribute	Description
Orange colour	Intensity of orange colour, from light yellow to dark red-orange.
Turbidity	Attribute of appearance relating to cloudiness of the oil.
Red pepper odour	The odour associated with red bell pepper fruits.
Red pepper aroma	The aroma associated with red bell pepper fruits.
Nutty odour	The odour associated with raw nuts.
Nutty flavour	The flavour associated with raw nuts.
Waxy	The aroma associated by candle wax.
Bitterness	Fundamental taste sensation elicited by caffeine, quinine.
Spiciness	Burning sensation coming from vegetables such as hot pepper.
Viscosity	Texture attribute related to resistance to flow.
Mouthcoating	Attribute related to the oil lingering in mouth after swallowing.

**Table 8:** Quantitative descriptive profile^®^ of four samples of pepper seed oil obtained by different extraction processes.

	P1-CP ^1^	P2-CP ^2^	P1-SC-CO_2_^3^	P2-SC-CO_2_^4^
Orange color **	67.25 ± 3.06 a	60.77 ± 3.62 ab	54.48 ± 3.04 b	58.56 ± 2.91 ab
Turbidity	4.45 ± 2.27 a	7.51 ± 4.25 a	4.05 ± 1.84 a	4.92 ± 2.34 a
Red pepper odor	59.11 ± 4.37 a	58.24 ± 4.19 a	50.74 ± 4.51 a	54.2 ± 4.39 a
Nutty odor	37.62 ± 5.54 a	34.29 ± 5.37 a	38.67 ± 4.48 a	38.6 ± 5.74 a
Biterness	10.55 ± 2.92 a	7.5 ± 1.76 a	15.28 ± 5.89 a	13.05 ± 4.69 a
Red pepper aroma *	55.88 ± 3.65 a	47.61 ± 4.13 ab	42.81 ± 5.44 b	45.78 ± 3.64 ab
Nutty flavor	28.05 ± 7.15 a	27.61 ± 6.82 a	28.77 ± 6.38 a	31.62 ± 7.1 a
Spiciness	30.89 ± 4.98 a	28.94 ± 5.04 a	29.88 ± 6.73 a	25.16 ± 5.25 a
Waxy	1.00 ± 0.32 a	1.43 ± 0.61 a	0.67 ± 0.41 a	0.87 ± 0.28 a
Viscosity	46.52 ± 4.86 a	43.37 ± 4.74 a	49.75 ± 4.11 a	46.13 ± 5.05 a
Mouthcoating *	29.31 ± 5.49 a	31.84 ± 5.72 ab	35.18 ± 5.3 b	34.75 ± 5.91 ab

Values are mean ± SD of two replicates; different letters within the same row indicate significant differences (*p* < 0.05); average values and st. dev. (** *p* < 0.01; * *p* < 0.05). ^1^ P1-CP—pepper seed oil of the Podravka variety produced by cold pressing. ^2^ P2-CP—pepper seed oil of the Slavonka variety produced by cold pressing. ^3^ P1-SC-CO_2_—pepper seed oil of the Podravka variety produced by supercritical CO_2_ extraction. ^4^ P2-SC-CO_2_—pepper seed oil of the Slavonka variety produced by supercritical CO_2_ extraction.

**Table 9:** Acceptability of pepper seed oil obtained by different extraction techniques (CP and SC-CO_2_) on two varieties of red pepper (P1 and P2).

	P1-CP	P2-CP	P1-SC-CO_2_	P2-SC-CO_2_
Acceptability *	8.1 ± 1.71 a	7.4 ± 1.92 b	6.8 ± 1.69 bc	6.5 ± 2.45 c

Values are mean ± SD of two replicates; Different letters within the same row indicate significant differences (*p* < 0.05); * *p* < 0.05.
